# Knowledge regarding applications of virtual reality- haptic and augmented reality technologies among dental healthcare professionals in Saudi Arabia: A cross-sectional study

**DOI:** 10.6026/973206300223629

**Published:** 2026-06-30

**Authors:** Nishath Sayed Abdul, Yara Faisal Albahijan

**Affiliations:** 1 1Department of OMFS and Diagnostic Sciences, College of Medicine and Dentistry, Riyadh Elm University, Riyadh, Saudi Arabia; 2 Department of Dental Hygiene, College of Applied Medical Sciences, Riyadh Elm University, Riyadh, Kingdom of Saudi Arabia

**Keywords:** Knowledge, awareness, augmented reality (AR), virtual reality (VR) haptic, dental health care professionals, dental hygienists

## Abstract

Technologies like virtual reality, haptic and augmented reality offer great potential for improving dental education, particularly in the preclinical development of manual skills and spatial awareness. Therefore, it is of interest to assess the knowledge and awareness of virtual reality (VR) - haptic and augmented reality (AR) technology among dental healthcare professionals of Saudi Arabia. Awareness regarding VR/AR was fairly high among the study sample, with 75.5% knowing about these technologies. 67% had not used VR/AR in clinical or teaching practice and over half had attended related training. Thus, we show significant gap between awareness and practical application, underscoring the need for curriculum integration and targeted training to facilitate adoption in dental practice.

## Background:

The phrase "extended reality" (XR) includes all technologies of virtual reality (VR), augmented reality (AR) and mixed reality (MR), which can change how we function. Additionally, they affect our interactions with each other and our environment, along with our perceptions and responses to occurrence [1]. Paul Milgram was the first who proposed the concept of a reality- virtual continuum in the year of 1994; nevertheless, because of technological constraints during that period, minimal progress occurred until the early 2010s, when fresh technological developments allowed the resurrection of the AR/VR machine [2]. Virtual reality (VR) is an innovative method that can merge input/output data and instructions, encompassing visual, auditory, olfactory, haptic and gustatory elements into a cohesive experience. Its immediate reaction to external input from the operator while concurrently modifying internal output to influence the operator creates a closed-loop human-machine interaction (HMI) [3]. Haptic interaction is crucial in VR systems for enabling realistic engagements between the operator and virtual objects, enhancing immersive experiences and being valuable for applications like virtual training and remote control [4 , 5]. Haptic sensation is described as gathering information through touch or interaction using the skin's tactile system, considered the most intricate sensation due to its dependence on tactile receptors located across the entire body. The concentration of sensory cells beneath the epidermis is approximately 100/cm^2^, enabling the detection of different types of external stimuli. This comprehensive sensing system is also ideal for imitation by artificial tactile interfaces in the HMI domain [6]. Augmented Reality can be described as the improvement of sensory experience by incorporating supplementary information that cannot be detected by the five senses [7]. Augmented Reality (AR) is a technology that integrates virtual data with the physical environment. The technological tools it employs consist of multimedia, 3D modeling, real-time tracking and registration, smart interaction, sensing and others. Its concept involves using computer-generated virtual content, including text, images, 3D models, music, video and more and integrating it into the real world after simulation. By doing so, both type of information support one another, leading to an improvement of the real world. Intelligent display technology, 3D registration technology and intelligent interaction technology form the essential technological circle of AR and significantly contribute to the advancement of AR [8]. AR technologies for the consumer market have now reached maturity across various potential application areas. The increasing number of publications on AR for surgery, medicine and rehabilitation indicates a strong demand for alternatives that can enhance current clinical practices in the healthcare sector [9]. The development of precise manual skills is necessary in dentistry, dentistry places strong emphasis on practical applications, especially the hands-on performance of restorative procedures, in contrast to many other health science fields [10]. Technologies like virtual reality and haptic, or force-feedback, stimulation offer great potential for improving dental education particularly in the preclinical development of manual skills and spatial awareness. The incorporation of haptic feedback technology in dental training corresponds with the changing educational environment, highlighting evidence-based methods and technology-oriented teaching [11]. Augmented- reality is distinguished from virtual reality (VR), which immerses the user in a computer-generated environment devoid of any tangible elements [12]. The interactive feedback enables with both these techniques provide students to enhance and improve their motor skills, hand-eye coordination and finesse while executing different dental procedures [13]. Therefore, it is of interest to assess the knowledge and awareness of virtual reality (VR) - haptic and augmented reality (AR) technology among dental healthcare professionals.

## Methodology:

A cross-sectional survey with 200 dental health care professionals was conducted from November 2025 to January 2026, after obtaining ethical approval from the institutional ethics committee (IRB Number - "FUGRP/2025/478/1382/1261") and informed consent from every participant in the research. A convenient sampling was used to drawn down the sample size of 200 and modified pre validated questionnaire that was split into four sections was utilized to evaluate demographics, knowledge, awareness and attitude towards application of VR-haptic and AR technologies among dental healthcare practitioners. The online survey was developed using Google Docs Form.

## Study tool:

The tool for data collection was a structured questionnaire, specifically tailored and modified for this study, based on previously published surveys assessing AR/VR knowledge in dental education, particularly the research conducted by Masood *et al.* and Ünal *et al.* [14, 15. A group of specialists evaluated the questionnaire to verify its suitability for all study subjects, including closed-ended (mainly dichotomous) response choices. The finished questionnaire comprised four sections:

[1] Section 1: demographic details which include age, gender and educational background.

[2] Section 2: knowledge related to virtual reality, virtual reality-haptic and augmented reality.

[3] Section 3: Awareness related with these technologies.

[4] Section 4: Attitude towards the application of these techniques in dentistry. Google forms used to conduct the final survey online.

## Data analysis:

Data collection evaluation responses were categorized and input into SPSS (version 29.0, IBM Corp., Armonk, NY, ARTICLE IN PRESS USA) for assessment. Descriptive statistics were computed for all variables; categorical data represented as frequencies and percentages. Comparison of attitude towards application among DHCP was performed by Chi- square test and p-value <0.05 considered significant.

## Results:

A total of 200 dental healthcare professionals participated in this study, with 96(48%) of them being male and 104 (52%) being female. The distribution by age group revealed that 30 participants (15%) belonged to the 18-22 age group, 56 participants (28%) to the 23-27 age group, 45 participants (22.5%) to the 28-32 age group and 69 participants (34.5%) to the above 32 age group. 30 (15%) were dental students, 47(23.5%) were dental interns, 24(12%) were postgraduates, 12(6%) were dental hygienist, 57(28.5%) were dental specialist and 30 (15%) were general dentist (Table 1). When knowledge of VR and AR technologies was evaluated in the current study, it was discovered that 151 DHCPs (75.5%) knew what these technologies were, while 49 (24.5%) did not and 48 (31.7%) knew how these technologies were used in dentistry. The prime source of knowledge about VR and AR among study participants was research article 56(37.8%) and 58(38.4%). When participants were asked about disadvantages of VR and AR technologies majority 108(54%) said that high cost/expensive (Table 2). In this study, when awareness regarding application of VR and AR technologies in dentistry was assessed, it was found that 110(55%) participants were aware about the application of VR and AR techniques in dental field, whereas awareness about the application of VR-Haptic in dentistry among DHCP was found 108(54%). When awareness on application of VR and AR technologies in dental education was assessed, it was found that 115(57.5%) and 104(52%) respectively in DHCP (Table 3). When DHCPS attitudes toward Virtual Reality-Haptic and Augmented Reality technologies in dentistry were evaluated, it was discovered that 152 participants (67%) did not use these, methods in clinical dentistry or in teaching. In this study, 105 DHCP (52.5%) attended workshops, seminars, or training sessions on VR- Haptic, while 104 DHCP (52%) attended work workshops, seminars, or training sessions on AR in healthcare. Additionally, 130 study participants, or 65% of the total, agreed to take part in CDE sessions related to these technologies. Regarding the choice between VR-Haptic and AR technology, 44 DHCP (22%) stated that both methods are useful in clinical and pedagogical dentistry (Table 4). A comparison of attitude towards the application of VR and AR technologies in clinical and education dentistry was compared among DHCPs revealed statistically significant difference was present among different dental health care providers (p<0.001)(Table 5).

## Discussion:

Many recent technological developments in the field of dentistry have altered the scope of numerous dental subspecialties. New innovations have been introduced to future improve and enhance dental education as well as the clinical application of dentistry. These include virtual reality (VR) and augmented reality (AR), which have been developed and researched with the intention of enhancing dentistry. A cross-sectional survey was conducted to assess the knowledge and awareness of virtual reality (VR)-haptic and augmented reality (AR) technology among dental healthcare professionals of Saudi Arabia. This study found that when knowledge about VR and AR technologies assessed it was found that 151 (75.5%) DHCP knows what is VR and AR technologies whereas 49(24.5%) don't know about these technologies. When comparison of knowledge was done among different dental health care professionals it was found that acquaintance was comparatively higher among specialty dentist. About 75% of participants in the current study were aware of these technologies, but their application in dentistry is low. Similarly, when Masood *et al.* conducted a study, a statistically significant difference of knowledge (1.40 ± 0.49) among different dental healthcare professionals was found and a paucity of knowledge and awareness among dental healthcare professionals about AR and VR technology in dentistry was discovered [14]. In this study, 54% of dental professionals believed that the main drawback of VR and AR technologies was their high cost; this could be the reason why they did not use techniques in their day-to-day clinical practice. Bencharit *et al.* also noted fewer negative aspects of using VR and AR technology, such as features that are absent or lacking, high overall cost, negative perceptions and lack of acceptance and the time needed for implementation [16]. In this study, only 48(31.7%) participants were aware about the application of VR and AR techniques in dental field. Whereas according to Ünal *et al.* only 20.9% of students said they had previously heard of AR/VR in dentistry and less than 1% had firsthand experience [15]. Over 88% of participants expressed a desire for virtual reality-based technology to be included in undergraduate curriculum, according to Antony *et al.* [17]. Over 92% of respondents stated that investing in new technologies is important or very important for success in dentistry, despite the fact that VRBT use and education are not included in their dental school curriculum [17]. An investigation carried out by de Boer *et al.* in Netherlands discovered that the haptic stimulation provides realistic tactile sensation in preclinical instruction and is realistic enough to replace the conventional phantom head [18]. When DHCPS attitudes toward virtual reality-haptic and augmented reality technologies in dentistry were evaluated, it was discovered that 152 participants (67%) did not use these, methods in clinical dentistry or in teaching. In this study, 105 DHCP (52.5%) attended workshops, seminars, or training sessions on VR- Haptic, while 104 DHCP (52%) attended work workshops, seminars, or training sessions on AR in healthcare. Additionally, 130 study participants, or 65% of the total, agreed to take part in CDE sessions related to these technologies. Attendances at relevant workshops or seminars was infrequent (3.1%), according to Ünal *et al.* despite this restricted exposure, 74.4% said they would be willing to take part in research and 83.8% said they would like to receive training [15]. In the present study, when DHCPs attitude toward the use of VR and AR technologies in clinical and educational dentistry were compared, it was found that there was a statistically significant difference between the various dental health care providers. Dental specialists had the most knowledge and application skills, followed by dental postgraduates, general dentist, dental interns, dental students and dental hygienists. Similarly, Masood *et al.* found that dental specialists showed a relatively higher level of awareness than their counterparts in other specialties. For technology adoption in dental education and practice to be successful, obstacles- most notably a lack of knowledge-must addressed [14]. Regarding the choice between VR-Haptic and AR technology, 44 DHCP (22%) stated that both methods are useful in clinical and pedagogical dentistry. In contrast, the majority of respondents (58%) in a study by Bencharit *et al.* believed that using VR-haptic in conjunction with phantom head training during the preclinical stage was the best strategy and this study came to the conclusion that VR- haptic technology is continuously developing and will probably gain acceptance as a crucial component of dental hand skill development to supplement conventional preclinical training [16]. To help students learn and practice using VR and AR technologies, the standard score method can be standardized. Numerous devices and system use VR and AR, such as Dent Sim TM, a comprehensive system that integrates VR and AR with ergonomic postures, immediate feedback, exam simulation, direct data transfer and campus usages [19, 20]. Student's and dentist positive attitudes toward dental simulation equipment are in line with studies conducted in Sweden, Switzerland, the United Kingdom and the Netherlands. These studies found that a significant portion of students were very satisfied with simulators tested, found this new method of learning to be motivation and had a positive attitude toward the introduction of simulation into the curriculum [21]. In addition to integrating learning and teaching systems from an organizational standpoint, the system with VR and AR also improves hand- eye coordination and training skills [22, 23]. When compared to conventional preclinical teaching methods, a system utilizing VR and AR can reduce faculty time by five times, making it an educational tool that allows students to learn on their own. Further application of these technologies is anticipated to improve their functionality and accessibility, which ultimately translate into a significant therapeutic or preventive impact.

## Conclusion:

We show that a lack of knowledge and awareness among dental healthcare professionals regarding VR, VR- Haptic and AR technology in dentistry was found. Dentistry is a challenging field that requires application skills and clinical knowledge. Virtual reality, augmented reality and haptic technologies have enormous potential to enhance dental education, especially in the preclinical development of manual skills and spatial awareness. Dental students can benefit from immersive, practical learning experiences offered by VR and AR, which can improve their comprehension and clinical skills.

## Figures and Tables

**Table 1:** Demographic details of study participants

**Demographic details**	**Number (%)**
**Age**	
1.18-22	30(15%)
2.23-27	56(28%)
3.28-32	45(22.5%)
4.32 above	69(34.5%)
**Gender**	
1.Male	96(48%)
2.Female	104(52%)
**Educational Level**	
1.Dental Students	30(15%)
2.Dental Interns	47(23.5%)
3.Dental Postgraduates	24(12%)
4.Dental Hygienist	12(6%)
5.Dental Specialist	57(28.5%)
6.General Dentist	30(15%)

**Table 2:** Knowledge based questions on applications of virtual reality-haptic and augmented reality technologies in dentistry

**Questions**	
Q1. Do you know about VR technology?	
Yes	151(75.5%)
No	49(24.5%)
If yes, do you know how it is applied in dentistry?	48(31.7%)
Yes	103(68.2%)
No	
Q2. Do you know about VR- Haptic technology?	
Yes	148(74%)
No	52(26%)
If yes, do you know how it is applied in dentistry?	
Yes	48(31.7%)
No	103(68.2%)
Q3. Do you know about AR technology?	
Yes	151(75.5%)
No	49(24.5%)
If yes, do you know how it is applied in dentistry?	
Yes	48(31.7%)
No	103(68.2%)
Q4. How do you know (source) about VR-Haptic technology?	
From newspaper	12(8.1%)
From research article	56(37.8%)
From my training	18(12.1%)
From professors/teachers	32(21.6%)
From colleagues/friends	18(12.1%)
Other sources	12(8.1%)
Q5. How do you know about AR technology?	
From newspaper	12(8.1%)
From research article	58(38.4%)
From my training	18(12.1%)
From professors/teachers	33(21.8%)
From colleagues/friends	18(12.1%)
Other sources	12(8.1%)
Q6. Do you know any disadvantages of VR technologies?	
High cost /expensive	108(54%)
Difficult to handle	28(14%)
Both A & B	32(16%)
I don't know	28(14%)
No disadvantages	4(2%)
Q7.Do you know any disadvantages of AR technologies?	
High cost /expensive	108(54%)
Difficult to handle	28(14%)
Both A & B	32(16%)
I don't know	28(14%)
No disadvantages	4(2%)

**Table 3:** Awareness based questions on Virtual Reality-Haptic and Augmented Reality Technologies in dentistry

**Questions**	
Q1. Are you aware of VR technology application in dental field?	
Yes	110(55%)
No	90(45%)
Q2. Are you aware of VR-Haptic technology application in dental field?	
Yes	108(54%)
No	92(46%)
Q3. Are you aware of AR technology application in dental field?	
Yes	110(55%)
No	90(45%)
Q4. Are you aware of applications of VR -haptic technologies in dental education or clinical practices in Saudi Arabia?	
Yes	115(57.5%)
No	85(42.5%)
Q5. Are you aware of applications of AR technologies in dental education or clinical practices in Saudi Arabia?	
Yes	104(52%)
No	96(48%)

**Table 4:** Attitude based questions on Virtual Reality- Haptic and Augmented Reality Technologies in dentistry

**Questions**	
Q1. Have you ever-used VR-haptic technology in your dental practice?	
Yes	48(24%)
No	152(67%)
Q2. Have you ever-used AR technology in your dental practice?	
Yes	48(24%)
No	152(67%)
Q3. Have you ever-used VR technology in education/teaching?	
Yes	48(24%)
No	152(67%)
Q4. Have you ever-used AR technology in education/teaching?	
Yes	48(24%)
No	152(67%)
Q5. Have you ever attended a workshop, seminar, or training session on uses of VR - haptic in healthcare?	
Yes	104(52%)
No	96(48%)
Q6. Have you ever attended a workshop, seminar, or training session on uses of AR in healthcare?	
Yes	105(52.5%)
No	95(47.5%)
Q7. Are you willing to participate in CDE and training programs related to VR-haptic in healthcare?	
Yes	130(65%)
No	70(35%)
Q8. Are you willing to participate in CDE and training programs related to AR in healthcare?	
Yes	130(65%)
No	70(35%)
Q9. If you are planning to apply in clinical practice these technologies, which one is better VR- Haptic technology or AR technology?	
VR	
AR	41(20.5%)
both	29(14.5%)
don't know	44(22%)
	86(43%)
Q10. Which method of learning is better VR / AR technologies/ traditional method?	
VR	41(20.5%)
AR	29(14.5%)
Both	44(22%)
don't know	86(43%)

**Table 5:** Comparison of application of VR and AR technologies among DHCP as per there educational level

**Question**	**Dental Students**	**Dental Interns**	**Dental Postgraduates**	**Dental Hygienist**	**Dental Specialist**	**General Dentist**	**P value**
Q1. Have you ever-used VR-haptic technology in your dental practice?							
Yes	0(0%)	2(1%)	12(6%)	0(0%)	31(15.5%)	03(1.5%)	0.001
No	30(15%)	45(22.5%)	12(6%)	12(6%)	26(13%)	27(13.5%)	
Q2. Have you ever-used AR technology in your dental practice?							
Yes	0(0%)	02(1%)	12(6%)	0(0%)	31(15.5%)	03(1.5%)	0.001
No	30(15%)	45(22.5%)	12(6%)	12(6%)	26(13%)	27(13.5%)	
Q3. Have you ever-used VR technology in education/teaching?							
Yes	0(0%)	02(1%)	12(6%)	0(0%)	31(15.5%)	03(1.5%)	0.001
No	30(15%)	45(22.5%)	12(6%)	12(6%)	26(13%)	27(13.5%)	
Q4. Have you ever-used AR technology in education/teaching?							
Yes	0(0%)	02(1%)	12(6%)	0(0%)	31(15.5%)	03(1.5%)	
No	30(15%)	45(22.5%)	12(6%)	12(6%)	26(13%)	27(13.5%)	
Q5. Have you ever attended a workshop, seminar, or training session on uses of VR- haptic in healthcare?							
Yes	6(3%)	19(9.5%)	14(7%)	4(2%)	41(20.5%)	20(10%)	0.001
No	24(12%)	28(14%)	10(5%)	08(4%)	16(8%)	10(5%)	
Q6. Have you ever attended a workshop, seminar, or training session on uses of AR in healthcare?							
Yes	02(1%)	23(11.5%)	21(10.5%)	4(2%)	44(22%)	11(5.5%)	0.001
No	28(14%)	24(12%)	03(1.5%)	08(4%)	13(6.5%)	19(9.5%)	
Q7. Are you willing to participate in CDE and training programs related to VR-haptic in healthcare?							0.902
Yes	18(9%)	31(15.5%)	17(8.5%)	04(2%)	35(17.5%)	20(10%)	
No	12(6%)	06(3%)	07(3.5%)	08(4%)	22(11%)	10(5%)	
Q8. Are you willing to participate in CDE and training programs related to AR in healthcare?							0.902
Yes	18(9%)	31(15.5%)	17(8.5%)	09(4.5%)	35(17.5%)	20(10%)	
No	12(6%)	06(3%)	07(3.5%)	03(1.5%)	22(11%)	10(5%)	
Q9. If you are planning to apply in clinical practice these technologies, which one is better VR- Haptic technology or AR technology?							
1	05(2.5%)	04(2%)	06(3%)	0(0%)	11(5.5%)	15(7.5%)	0.001
2	0(0%)	05(2.5%)	05(2.5%)	0(0%)	11(5.5%)	08(4%)	
3	05(2.5%)	11(5.5%)	05(2.5%)	05(2.5%)	18(9%)	0(0%)	
4	20(10%)	27(13.5%)	08(4%)	07(3.5%)	17(8.5%)	07(3.5%)	
Q10.Which method of learning are better VR / AR technologies/ traditional method?							
1	05(2.5%)	04(2%)	06(3%)	0(0%)	11(5.5%)	15(7.5%)	0.001
2	0(0%)	05(2.5%)	05(2.5%)	0(0%)	11(5.5%)	08(4%)	
3	05(2.5%)	11(5.5%)	05(2.5%)	05(2.5%)	18(9%)	0(0%)	
4	20(10%)	27(13.5%)	08(4%)	07(3.5%)	17(8.5%)	07(3.5%)	
